# Refractory aphthae in the epiglottis and supraglottic area due to Crohn's disease

**DOI:** 10.1002/jgf2.356

**Published:** 2020-06-28

**Authors:** Hiroshi Hori, Takahiko Fukuchi, Hitoshi Sugawara

**Affiliations:** ^1^ Division of General Medicine Department of Comprehensive Medicine 1 Saitama Medical Center Jichi Medical University Saitama Japan

**Keywords:** Crohn's disease, epiglottis, fever of unknown origin, refractory aphthous ulcers

## Abstract

A 27‐year‐old woman complaining of persistent fever and sore throat was consulted. Examination of the laryngopharynx revealed multiple aphthous ulcers in the uvula, arytenoids, epiglottis, and laryngeal pyriform fossa. Crohn's disease was diagnosed by colonoscopy.

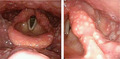

A 27‐year‐old woman consulted her local physician complaining of persistent fever (38°C), sore throat, and mild painful swallowing that had persisted for 1 month. She was referred to the Department of Otolaryngology at our hospital. Examination of the laryngopharynx revealed multiple aphthous ulcers in the uvula, arytenoids, epiglottis, and laryngeal pyriform fossa (Figure 1). Aphthous ulcers were concentrated in the larynx and particularly in the structures of the supraglottic area. Blood tests indicated inflammation (white blood cell count 14 630/μL [neutrophil 84%], C‐reactive protein [CRP] 12.25 mg/dL). Abdominal contrast‐enhanced computed tomography revealed edematous changes in the large intestine suggestive of Behcet's disease or inflammatory bowel disease leading to the performance of colonoscopy.

**Figure 1:**
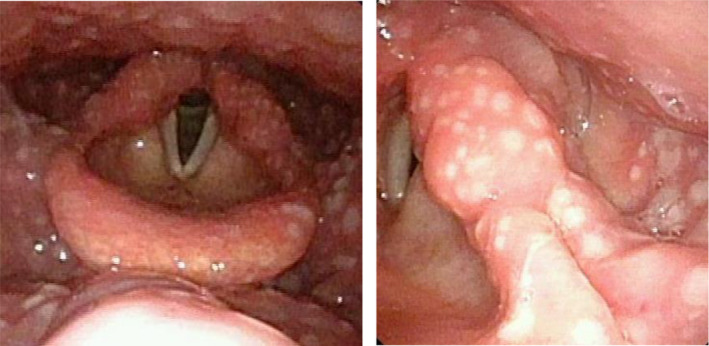
Multiple aphthous ulcers in the epiglottis and structures in the supraglottic area (the arytenoids, vallecula of epiglottis, and laryngeal pyriform fossa)

Multiple skip lesions were observed in the cecum and colon, and a longitudinal ulcer was observed in the transverse colon (Figure 2). Noncaseating granulomas were not identified; however, histological findings were consistent with Crohn's disease (CD). The patient developed no skin rashes, pubic ulcers, and eye lesions and was the first oral aphthae. Based on these findings, the CD of the large bowel was diagnosed.

**Figure 2:**
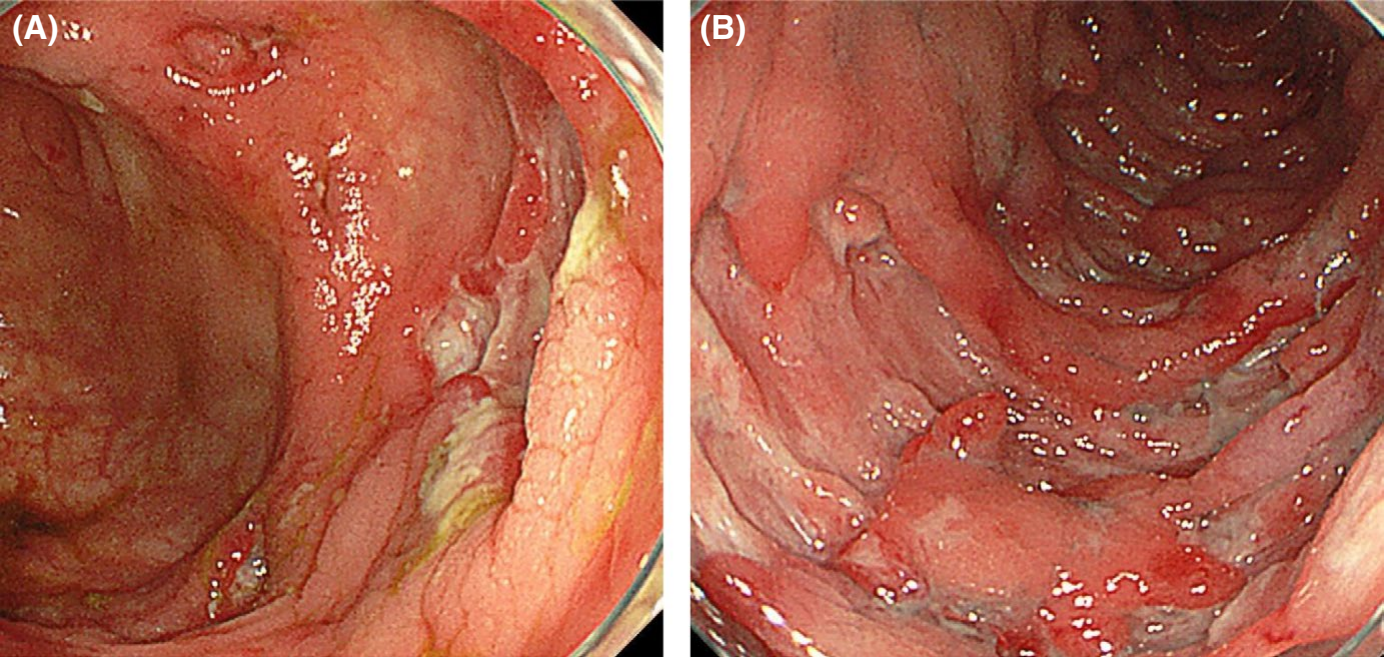
Ulcerative lesions in the cecum (A) and a longitudinal ulcer in the transverse colon (B)

The patient was administered 40 mg (1.0 mg/kg) prednisolone, leading to rapid improvement in the aphthous laryngeal ulcers and CRP levels normalized. Throughout the course of her illness, the patient complained of soft stools but experienced no abdominal pain or melena.

In patients with refractory aphthous ulcers in the epiglottis, arytenoids, and pyriform fossa, it is important to suspect CD. CD is chronic disease that can cause granulomatous inflammation in any part of the gastrointestinal tract from the mouth to the anus. However, laryngeal involvement is uncommon and occurs in only 0.5%–13% of cases, usually in young men.^1^ Symptoms of laryngeal lesions in CD include painful swallowing, voice change, chronic cough, and dysphagia, with swelling and ulceration of the supraglottic area, including the epiglottis and arytenoids, and limited vocal fold mobility observed by endoscopy.^1^

Oral and laryngeal CD spontaneously improves in 25% of cases irrespective of the gastrointestinal lesions activity. In 26%‐60% of cases, they can precede gastrointestinal symptoms, thereby rendering diagnosis difficult.^2^ CD should be considered in patients with repeated aphthous lesions of the larynx and pharynx. In addition to CD, differential diagnosis of ulcers in the laryngopharynx area includes Behcet's disease, tuberculosis, syphilis, fungal infection, sarcoidosis, Wegener's granulomatosis, gastroesophageal reflux disease, IgG4RD, and lymphoma.^1^ To differentiate between these diseases, the presence or absence of lesions other than those of the laryngopharynx (such as, rash, arthritis, gastrointestinal symptoms, and eye symptoms) and pathology findings is important; however, in actual practice, differential diagnosis is often difficult. Reliance on pathology findings of noncaseating granuloma can be challenging as their presence varies from 10% to 75% depending on the report.^3^ However, considering the distribution of the lesions, only a few reports of lesions of the epiglottis were discovered, and these were limited to CD and Behcet's disease, supporting our diagnosis.^4^, ^5^

In conclusion, when refractory repeated aphthous ulcers are observed in the epiglottis and supraglottic area, CD or Behcet's disease should be considered.

## CONFLICT OF INTEREST

The authors have stated explicitly that there are no conflicts of interest in connection with this article.

## INFORMED CONSENT

We have obtained the consent of the patient for publication.
